# Feasibility of laparoscopy for small bowel obstruction

**DOI:** 10.1186/1749-7922-4-3

**Published:** 2009-01-19

**Authors:** Eriberto Farinella, Roberto Cirocchi, Francesco La Mura, Umberto Morelli, Lorenzo Cattorini, Pamela Delmonaco, Carla Migliaccio, Angelo A De Sol, Luca Cozzaglio, Francesco Sciannameo

**Affiliations:** 1 Department of General and Emergency Surgery, St Maria Hospital, Terni, University of Perugia, Perugia, Italy; 2 Department of Surgical Oncology, IRCCS Istituto Clinico Humanitas, Rozzano, Milan, Italy

## Abstract

**Background:**

Adherential pathology is the most common cause of small bowel obstruction. Laparoscopy in small bowel obstruction does not have a clear role yet; surely it doesn't always represent only a therapeutic act, but it is always a diagnostic act, which doesn't interfere with abdominal wall integrity.

**Methods:**

We performed a review without any language restrictions considering international literature indexed from 1980 to 2007 in Medline, Embase and Cochrane Library. We analyzed the reference lists of the key manuscripts. We also added a review based on international non-indexed sources.

**Results:**

The feasibility of diagnostic laparoscopy is high (60–100%), while that of therapeutic laparoscopy is low (40–88%). The frequency of laparotomic conversions is variable ranging from 0 to 52%, depending on patient selection and surgical skill. The first cause of laparotomic conversion is a difficult exposition and treatment of band adhesions. The incidence of laparotomic conversions is major in patients with anterior peritoneal band adhesions. Other main causes for laparotomic conversion are the presence of bowel necrosis and accidental enterotomies. The predictive factors for successful laparoscopic adhesiolysis are: number of previous laparotomies ≤ 2, non-median previous laparotomy, appendectomy as previous surgical treatment causing adherences, unique band adhesion as phatogenetic mechanism of small bowel obstruction, early laparoscopic management within 24 hours from the onset of symptoms, no signs of peritonitis on physical examination, experience of the surgeon.

**Conclusion:**

Laparoscopic adhesiolysis in small bowel obstruction is feasible but can be convenient only if performed by skilled surgeons in selected patients. The laparoscopic adhesiolysis for small bowel obstruction is satisfactorily carried out when early indicated in patients with a low number of laparotomies resulting in a short hospital stay and a lower postoperative morbidity. Although a higher small bowel obstruction recurrence remains the major postoperative risk of the laparoscopic management of these patients.

## Background

The small bowel is the most frequent intestinal occlusion site and adherential pathology represents the most common cause of small bowel obstruction (80%) [1]. Other less common causes are: peritoneal carcinosis, Crohn disease, GIST, internal hernia, diaphragmatic hernia, Meckel's diverticulum, and biliary ileus [1].

Laparoscopy in small bowel obstruction has not a clear role yet; surely it is a diagnostic act and sometimes also a therapeutic act, which does not interfere with abdominal wall integrity [2,3].

The first laparoscopic adhesiolysis for small bowel obstruction was performed by Mouret in 1972 [4]. Following this first case, the use of laparoscopy for treating small bowel obstruction was accepted by other surgeons and the indication was represented by patients with unique band adhesion and no clinical signs of bowel ischemia or necrosis [5].

In laparoscopic adhesiolysis for small bowel obstruction the first trocar needs to be placed using Hasson's technique for open laparoscopy in order to avoid accidental bowel perforations related to bowel distension and adhesions with the abdominal wall. Two 5 mm trocars must be introduced under vision in order to explore the peritoneal cavity. Dilated bowels are moved away to find out the obstructed bowel segment by the band adhesion. If the surgeon notices ischemic or necrotic bowel he performs a laparotomy, on the contrary if the bowel appears healthy the laparoscopic procedure can be delivered and an atraumatic grasp can be used to isolate the band adhesion, which is coagulated by bipolar coagulator and then sectioned with scissors. These manoeuvres result in the liberation of the obstructed small bowel segment.

In order to perform an emergency laparoscopic adhesiolysis, three factors are fundamental:

• Early indication for surgical treatment.

• Exclusion of patients with history of multiple abdominal surgical procedures.

• Exclusion of patients with suspected strangulation or small bowel torsion associated with ischemic or necrotic bowel.

It is often not possible to achieve a preoperative diagnosis of mechanical small bowel obstruction caused by peritoneal adherences [6]. For this reason the number of patients and the quality of the studies published in literature on this topic are both low, resulting in poor scientific evidences. The first review concerning laparoscopic adhesiolysis of the small bowel obstruction was written by Reissman and Wexner [7]. The following reviews were by Duron [8] and Slim [9] in 2002 and Nagle [10] in 2004. In 2006 *Société Française de Chirurgie Digestive *(SFCD) published a review [3] from which evidence-based recommendations could be extracted. In this review, because of absence of randomized studies in literature, the Authors considered only 11 studies with a minimum of 40 patients, of which 3 were perspectives and 2 with a patient group treated by laparotomic surgery. In the same years European Association for Endoscopic Surgery (EAES) guidelines for the laparoscopic treatment of abdominal emergencies [11] were also published, and three other reviews were realized by Darzi [12], Tsumura [13] and Majewsky [14].

The aim of this paper is to analyse the feasibility and convenience of the laparoscopic adhesiolysis suggesting the successful predictive factors and the absolute and relative contraindications, which lead to an accurate selection of patients resulting in a lower postoperative morbidity.

## Methods

We performed a review, considering international literature indexed in Medline, Embase and Cochrane Library without any language restrictions, from 1980 to 2007. The literature searches were carried out using the following keywords: "laparoscopic adhesiolysis", "laparoscopic lysis", "laparoscopic management", "AND small bowel obstruction", "AND adhesive bowel obstruction".

Furthermore we analysed other non-indexed sources: records from the congresses of *Società Italiana di Chirurgia *(SIC) and *Associazione Chirurghi Ospedalieri Italiani *(ACOI), records from *Association Française de Chirurgie *(AFC), Eastern Europe online surgical journals (*Chirurgia *and *Jurnalul de Chirurgie*), Spanish online surgical journals *(Cirurgia Espanola *and *Anales del sistema sanitario de Navarra*), and online specialized journals dedicated to adherential pathology (Adhesions).

Studies including a small number of patients (<5) treated with emergency laparoscopic adhesiolysis or patients treated electively for adherential syndrome were excluded from our review.

## Results and discussion

This literature research pointed out different studies (Table 1) [6,15-44] confirming the main diagnostic role of laparoscopic adhesiolysis. In fact the mentioned studies show that while the feasibility of diagnostic laparoscopy is high (60–100%), that of therapeutic laparoscopy is low (40–88%).

**Table 1:** Laparoscopic management of small bowel obstruction.

	**Emergency treated patients**	**Achived diagnosis (site and cause of occlusions)**	**Laparotomic conversions**
**Dallemagne **[6]	86	100%	23%

**Strickland **[15]	35	60%	37%

**Ibrahim **[16]	25	100%	28%

**Iorgulescu **[17]	6	100%	16,6%

**Benoist **[18]	31	**	48,4%

**Wullstein **[19]	52	**	51,9%

**Chopra **[20]	34	**	32,3%

**Saudemont **[21]	34	100%	50%

**Kirshtein **[22]	44	97%	25%

**Bailey **[23]	55	**	16,3%

**Borzellino **[24]	40	**	25%

**Levard **[25]	23	**	52,1%

**Parent **[26]	30	**	30%

**Chèvre **[27]	20	**	35%

**Suter **[28]	71	78%	35,2%

**Khaikin **[29]	31	100%	32%

Multicenter F.A.S.R.* [30]	261	**	37,5%

**Hoyuela **[31]	10	94,4%	0

**Navez **[32]	54	66%	48,2%

**Cavaliere **[33]	44	91%	23%

**Meinero **[34]	39	97,5%	12,8%

**Al-Mulhim **[35]	9	100%	11,1%

**Liauw **[36]	5	100%	20%

**Johanet **[37]	49	**	34.7%

**Zerey **[38,39]	52	100%	16,7%

**Sciannameo **[40]	27	100%	11,1%

**Chosidow **[41]	39	**	36%

**Bergamini **[42]	13	**	46,1%

**El Dahha **[43]	13	**	7,6%

**Binenbaum **[44]	4	**	50%

* F.A.S.R. French Association for Surgical Research

** Not indicated by the Authors

The main evidence-based recommendation from the SFCD [3] and EAES [11] was: "It is not possible to recommend (EL 4) laparoscopic adhesiolysis as an alternative to the laparotomic approach for small bowel obstructions (C grade)".

The same conclusion comes from Darzi's review [12]. Also in the Slim's study the conclusions are comparable to SFCD and EAES, besides the Author individuated a patient subgroup previously treated with appendectomy in which laparoscopic approach is feasible and convenient (D grade) [9,45].

Duron [8], Nagle [10], Tsumura [13], Majewski [14] and Perniceni [46] stated that laparoscopic adhesiolysis is feasible and convenient only if performed by skilled surgeons on selected patients.

### Feasibility and convenience of laparoscopic adhesiolysis

Basic technical needs for performing laparoscopic adhesiolysis are good surgical skills, the open laparoscopy approach [15-20] and the possibility to move the operating table in different positions in order to point out the adherences [4,21,47-51]. In this review the evaluation of feasibility of laparoscopic adhesiolysis was made considering and analyzing the frequency of two major events, the laparotomic conversions and the relapse of small bowel obstruction.

The frequency of laparotomic conversions is variable ranging from 0 to 52% (Table 1) [6,15-44], depending on patient selection and surgical skill [45]. In order to reduce the number of conversions some surgeons perform a hand-assisted laparoscopy in some selected cases [22,23,52]. The first cause of laparotomic conversion is a difficult exposition and treatment of band adhesions (Table 2) [15,16,18-22,24-27,29,38,39,41,42]; this is due to a reduced operating field caused by small bowel dilatation [24,46], multiple band adhesions [22], and occasionally by the presence of posterior peritoneal band adhesions [13], which are more difficult to treat laparoscopically.

**Table 2:** Causes of laparotomic conversions.

		**Causes of laparotomic conversions**		
		
	**Patients with laparotomic conversion**	**Difficult exposition/treatment of band adhesions**	**Bowel necrosis**	**Accidental enterotomies**
**Strickland **[15]	13	69,23%	15,38%	23%

**Ibrahim **[16]	11	27,2%	9%	18,1%

**Benoist **[18]	15	33,4%	20%	0

**Wullstein **[19]	27	37%	37%	25,9%

**Chopra **[20]	11	72,6%	9%	36.3%

**Saudemont **[21]	17	52,9%	35,3%	11,8%

**Kirshtein **[22]	11	72,7%	0	27,3%

**Borzellino **[24]	10	80%	10%	10%

**Levard **[25]	12	58,3%	8,4%	33,3%

**Parent **[26]	9	66,6%	0	33,3%

**Chèvre **[27]	7	85,7%	0	14,3%

**Khaikin **[29]	10	50%	40%	0

**Zerey **[38,39]	4	100%	0	0

**Chosidow **[41]	14	28,57%	28,57%	14,28%

**Bergamini **[42]	6	66,7%	16,7%	0

In some cases it is necessary to use one or two additional 5 mm trocars to manipulate the bowel and point out the band adhesions. If these adhesions are not visible, a laparotomic conversion is necessary. Sometimes, the main band adhesion causing obstruction is not pointed out, and only those band adhesions which are easier to remove get resected. In this case the obstruction persists, and the patient will need a laparotomy for treating the incomplete laparoscopic adhesiolysis [46]. Tsumura [13] classified the different location of obstructive band adhesions and estimated their frequency: anterior visceroparietal adhesions (between anterior abdominal wall and small bowel) (40%), anterior visceroparietal adhesions associated to viscerovisceral adhesions (small bowel) (32%), viscerovisceral adhesions (small bowel) (16%), posterior visceroparietal adhesions (between posterior peritoneum and small bowel) (8%), anterior and posterior visceroparietal adhesions associated to viscerovisceral adhesions (4%). The incidence of laparotomic conversions is major in patients with anterior peritoneal band adhesions (anterior visceroparietal adhesions, anterior visceroparietal adhesions associated to viscerovisceral adhesions and viscerovisceral adhesions) compared to patients with posterior band adhesions (posterior visceroparietal adhesions, anterior and posterior visceroparietal adhesions associated to viscerovisceral adhesions) (50% vs 22.7%).

Other main causes for laparotomic conversion are the presence of bowel necrosis, which always needs a resection imperatively performed laparotomically [46,53], and accidental enterotomies.

The frequency of accidental enterotomies is variable (Table 2) [15,16,18-22,24-27,29,38,39,41,42], being more frequent in patients who have a history of previous multiple laparotomies [3,19]. Most of the accidental enterotomies occur while performing adhesiolysis. The other less common mechanism of injury is the Verres needle insertion, reported in the Levard's [25], Parent's [26] and Chèvre's [27] series. It is often necessary to perform a laparotomic conversion in order to suture or to perform a resection and anastomosis of the perforated bowel. The suture performed through open access gives more chances of endurance and safety, especially when done on a dilated and fragile obstructed bowel [54]. When the accidental enterotomy is not pointed out at operating time, it can show up in postoperative course as a peritonitis that increases morbidity and mortality. Unrecognized accidental enterotomies, discovered by the onset of postoperative peritonitis, are an increasingly frequent cause of malpractice claims [55].

Defensive medicine has delineated many practical strategies in order to avoid accidental enterotomies during laparoscopic adhesiolysis: accurate patient selection excluding patients with history of multiple abdominal surgical procedures and taking early indication for surgical treatment, and particular attention to surgical techniques [56] always staying close to parietal peritoneum during dissection, not sectioning tenacious band adhesions and always controlling the direction of the instruments. Borzellino routinely performs a preoperatory ultrasonographic mapping of visceroparietal adhesions, in order to avoid lesions resulting from Veress' needle insertion [24].

In the tables 3 and 4 we report the predictive factors for successful laparoscopic adhesiolysis and the absolute and relative contraindications to laparoscopic adhesiolysis, which allow performing an accurate selection of patients with small bowel occlusion.

**Table 3:** Predictive factors for successful laparoscopic adhesiolysis.

• Number of previous laparotomies ≤ 2 [8,9,46,57]
• Non-median previous laparotomy [9,45,46]
• Appendectomy as previous surgical treatment causing adherences [11,17,28,46]
• Unique band adhesion as pathogenetic mechanism of small bowel obstruction [8,46,57]
• Early laparoscopic management within 24 hours from the onset of symptoms) [8,11,28,46,57]
• No signs of peritonitis on physical examination [24,46,49]
• Experience of the surgeon [46,49,58]

**Table 4:** Absolute and relative contraindications to laparoscopic adhesiolysis.

**Absolute contraindicaions**	**Relative contraindicaions**
• Abdominal film showing a remarkable dilatation (> 4 cm) of small bowel [3,10,11,24,28,49,58]	• Number of previous laparotomies > 2 [3,11,18,27,46]
• Signs of peritonitis on physical examination [3,18,58]	• Multiple adherences [3,18]
• Severe comorbidities: cardiovascular, respiratory and hemostatic disease [3,18,58]	
• Hemodynamic instability [58]	

Since the number of laparotomies is correlated to the grade of adherential syndrome, a number of previous laparotomies ≤ 2 [8,9,46,57] is considered a predictive successful factor. As well, a non-median previous laparotomy [9,45,46] (McBurney incision), appendectomy as previous surgical treatment causing adherences [11,17,28,46], and a unique band adhesion as pathogenetic mechanism of small bowel obstruction [8,46,57] are predictive successful factors. On the other hand a number of previous laparotomies > 2 [3,11,18,27,46], and the presence of multiple adherences [3,18] can be considered relative contraindications. Furthermore since the presence of ischemic or necrotic bowel is an indication to perform a laparotomy, the absence of signs of peritonitis on physical examination [24,46,49] is another predictive successful factor, as it is very uncommon to find out an intestinal ischemia or necrosis without signs on clinical examination. Whereas their presence [3,18,58] is an absolute contraindication to laparoscopy because in case of peritonitis an intestinal resection and anastomosis could be needed and safely performed through open access. Another predictive factor is the early laparoscopic management within 24 hours from the onset of symptoms [8,11,28,46,57], before the small bowel dilatation reduces the laparoscopic operating field. For this reason an abdominal film showing a remarkable dilatation (> 4 cm) of small bowel [3,10,11,24,28,49,58] is an absolute contraindication. Other absolute contraindications are severe comorbidities, as cardiovascular, respiratory and hemostatic disease [3,18,58], and the hemodynamic instability [58], because they do not allow a safe pneumoperitoneum and need a brief surgical time. Obviously the experience of the surgeon [46,49,58] also influences the outcome of the laparoscopic adhesiolysis.

Laparotomic conversion is often related to a higher morbidity rate, for this reason it is necessary to evaluate a primary laparotomic access in those cases without predictive factors for successful adhesiolysis.

To shorten the operating time and reduce the laparotomic conversion rate, some surgeons suggest performing, when possible, a mini-laparotomy near the occlusion site detected laparoscopically [15,16,22,59]. Tsumura states that conversion through a mini-laparotomy still allows a mini-invasive access, with a shorter hospital stay (4.5 days in laparoscopically treated patients compared to 6.9 days in patients with a mini-laparotomic access, or 14 days in a patient treated by a classical laparotomic approach) [13,59]. As well Wexner considers more advantageous the video-assisted approach than laparotomic access. Although these advantages are more evident with the laparoscopic access rather than with the video-assisted approach: shorter operative time (75 min. laparoscopic treatment vs 98 min laparoscopy-assisted approach), postoperative hospital stay (4 vs 6,5 days), first bowel movement (3 vs 4 days) [29].

It is almost impossible to predict in the preoperatory phase if the obstruction is caused by a single band adhesion or by multiple adhesions [5]; some surgeons and radiologists state that a CT scan can help to determine the cases in which it is likely to be a large adhesion site blocking the bowel or causing intestinal necrosis [60,61], and which should be managed laparotomically.

The analysis of the convenience of laparoscopic adhesiolysis in small bowel obstructions was evaluated by using the following parameters: surgical operating time, hospital stay, morbidity, mortality and the bowel obstruction recurrence rate (Table 5) [19,29].

**Table 5:** Comparison between laparoscopic and laparotomic management of small bowel obstructions.

	**Laparoscopic management**		**Laparotomic management**	
	**Wullstein **[19]	**Khaikin **[29]	**Wullstein **[19]	**Khaikin **[29]

**Surgical operating time**	103 min	78 min	84 min	70 min

**Hospital stay **(postoperative)	11,3 days	5 days	18,1 days	9 days

**First bowel movement**	**	3 days	**	6 days

**Oral re-intake**	5,1 days		6,4 days	

**Morbidity**	19%	16%	40,4%	45%

**Bowel obstruction recurrence**	0–14,2%		0–4,6%	

** Not indicated by the Authors

The surgical operating time is greater in patients who underwent laparoscopic surgery compared to patients who underwent a laparotomy [19,29]. However the duration of laparoscopic procedure is variable ranging from 20 minutes for a simple band adhesion to 2–3 hours for more complex cases [62,63].

The hospital stay is shorter compared to a laparotomic approach [3,11,19,29,30], with an early flatus and early realimentation [19,29]. This is due to a short period of ileum paralysis following the laparoscopic adhesiolysis compared to the laparotomic procedure.

The postoperative morbidity is lower in patients who underwent laparoscopic adhesiolysis compared to those who underwent the laparotomic approach [19,29]. Furthermore a greater rate of morbidity is present in patients who underwent laparotomic conversion [19,29]; whereas mortality is comparable in the two groups (0–4%) [19,29].

Finally the laparoscopic adhesiolysis can avoid laparotomy, which is itself a cause of new adhesions and bowel obstruction [5,8,25,45,46], although some authors noticed a greater incidence of recurrent small bowel obstructions in patients who underwent laparoscopy compared to those in which a laparotomy was performed [3,30,52,62]. Duron attributes these contrasting results to the selection bias of the populations examined in different studies [31,57].

## Conclusion

Laparoscopic adhesiolysis in small bowel obstruction is feasible but can be convenient only if performed by skilled surgeons in selected patients. Performing an accurate selection of obstructed patients is essential in order to avoid an increase in morbidity due to laparotomic conversion. This review suggests the predictive factors for achieving this result, considering the number and kind of previous laparotomies, the previous surgical treatment causing adherences and grade of adherential syndrome, the time from the onset of obstructive symptoms and grade of intestinal dilatation on X-ray investigations, the association with intestinal ischemia or necrosis and consequent signs of peritonitis, the grade of the comorbidities and the hemodynamic condition.

The convenience of laparoscopic management of the correctly selected patients with small bowel obstruction is demonstrated, despite of a longer surgical operating time, by the short hospital stay, the early oral intake and especially by the lower postoperative morbidity. On the other hand the main disadvantage is the increased small bowel obstruction recurrence; furthermore the mortality rate remains unmodified.

Definitively the laparoscopic adhesiolysis for small bowel obstruction is satisfactorily carried out when early indicated in patients with a low number of laparotomies resulting in a short hospital stay and a lower postoperative morbidity. Although a higher small bowel obstruction recurrence remains the major postoperative risk of the laparoscopic management of these patients.

## Competing interests

The Authors state that none of the authors involved in the manuscript preparation has any conflicts of interest towards the manuscript itself, neither financial nor moral conflicts. Besides none of the authors received support in the form of grants, equipment, and/or pharmaceutical items.

## Authors' contributions

All authors contributed equally to this work.
